# Preclinical Efficacy of Novel Vesicular Monoamine Transporter 2 Inhibitors as Antagonists of d-Methamphetamine Self-Administration in Rats

**DOI:** 10.4172/2329-6488.1000e127

**Published:** 2015-10-27

**Authors:** Takato Hiranita

**Affiliations:** Division of Neurotoxicology, National Center for Toxicological Research (NCTR), U.S. Food and Drug Administration (FDA), 3900 NCTR Road, Jefferson, AR 72079-9501, USA

## Editorial

A series of studies by Drs. Linda Dwoskin and Michael Bardo demonstrated the preclinical efficacy of novel vesicular monoamine transporter 2 (VMAT2) inhibitors as antagonists of d-methamphetamine self-administration in rats [1-6]. This is an important finding since there is a lack of FDA-approved medications to treat amphetamine-type stimulant abuse. There are also few if any candidate compounds that show preclinical efficacy as amphetamine antagonists (e.g. [7]).

Reinforcing effects of stimulants result from their common capacity to increase extracellular dopamine (DA) levels in terminal regions of mesolimbic dopaminergic neurons [8]. Amphetamines are substrates for the dopamine transporter (DAT), while cocaine inhibits DA uptake and functions as a DAT inhibitor [8]. Thus stimulants function as indirect DA agonists. In addition to the DAT, uptake of amphetamines into cytoplasm via DAT results in DA release into synaptic clefts through actions at the cytoplasmic vesicular monoamine transporter 2 (VMAT2) in the brain [8]. Thus VMAT2 is a potential target of action for amphetamines. Consistent with this hypothesis, Drs. Dwoskin and Bardo demonstrated that novel VMAT2 inhibitors can decrease d-methamphetamine self-administration in rats [1-6]. Importantly, the d-methamphetamine-antagonist effects of VMAT2 inhibitors were specific for the reinforcing effects of d-methamphetamine. For example, a VMAT2 inhibitor N-(1,2R-dihydroxylpropyl)-2,6-cis-di-(4-methoxyphenethyl)piperidine hydrochloride (GZ-793A) was more potent in decreasing self-administration responding for d-methamphetamine than in decreasing that of cocaine [5] or food-reinforced responding [5,6]. The pharmacological specificity relative to food-reinforced responding was demonstrated with other novel VMAT2 inhibitors lobelane [4], meso-transdiene [3], and cis-2,5-di-(2-phenethyl)-pyrrolidine hydrochloride (UKCP-110) [1]. In addition, another group previously demonstrated a lack of effect for the prototype VMAT2 inhibitor reserpine on cocaine self-administration using rhesus monkeys [9]. In contrast, the prototype VMAT2 inhibitor (±)-tetrabenazine failed to exhibit pharmacological specificity. (±)-Tetrabenazine was equipotent in decreasing self-administration responding for d-methamphetamine and food reinforced responding [2].

Te novel VMAT2 inhibitors possess a clinically preferential profile since the duration of action as d-methamphetamine antagonists in vivo lasted at least 60 minutes [1-6], which is approximately 12-fold longer than the elimination half-life of the prototype VMAT2 inhibitor (±)-tetrabenazine [10]. However, the novel VMAT2 inhibitors need improvement to be useful clinically since they possess relatively low affinity for VMAT2 (Ki values >2,000 nM, see Table 1). VMAT2 is a cytoplasmic protein and VMAT2 inhibitors need to penetrate plasma membranes in vivo.

Despite the fact that the novel VMAT2 inhibitors exhibited low affinities for VMAT2, the series of studies by Drs. Dwoskin and Bardo demonstrated the preclinical efficacy of a novel class of antagonists for d-methamphetamine self-administration. Although it is still relatively unknown how amphetamines increase DA levels in synaptic clefts, these findings suggest that development of VMAT2 inhibitors as a specific amphetamine antagonists in vivo is possible.

## Figures and Tables

**Table 1:** Inhibition by various compounds of specific binding to the VMAT2 (K_i_ Value, nM).

Compound	VMAT2 ([^3^H] dihydrotetrazenazine binding)
(±)-Tetrabenazine	13 (± 1) [11]
GZ-793A	8,290 (± 2,790) [12]
Lobelane	2,040 (± 640) [13] 970 (± 190) [1]
Meso-Transdiene	9,880 (± 2,220) [14]
UKCP-110	2,660 (± 366) [1]
*d*-Methamphetamine	80,100 (± 19,500) [13] No inhibition at 100 μM [15]
*d*-Amphetamine	No inhibition at 100 μM [15]
Cocaine	No inhibition at 100 μM [16]
